# The genome sequence of the silverweed cinquefoil, *Potentilla anserina* L., 1753

**DOI:** 10.12688/wellcomeopenres.19908.1

**Published:** 2023-10-13

**Authors:** Maarten J. M. Christenhusz, Ilia J Leitch, Royal Botanic Gardens Kew Genome Acquisition Lab, Plant Genome Sizing collective, Darwin Tree of Life Barcoding collective, Wellcome Sanger Institute Tree of Life programme, Wellcome Sanger Institute Scientific Operations: DNA Pipelines collective, Tree of Life Core Informatics collective, Darwin Tree of Life Consortium

**Affiliations:** 1 Royal Botanic Gardens Kew, Richmond, England, UK

**Keywords:** Potentilla anserina, Argentina anserina, silverweed cinquefoil, genome sequence, chromosomal, Rosales

## Abstract

We present a genome assembly from a specimen of *Potentilla anserina* (the silverweed cinquefoil; Streptophyta; eudicotyledons; Rosales; Potentilleae). The haploid genome sequence is 237 megabases in span. Most of the assembly is scaffolded into seven chromosomal pseudomolecules. The mitochondrial and plastid genomes have also been assembled and are 294.6 and 155.6 kilobases in length respectively.

## Species taxonomy

Eukaryota; Viridiplantae; Streptophyta; Embryophyta; Tracheophyta; Spermatophyta; Magnoliopsida; eudicotyledons; Gunneridae; Pentapetalae; rosids; fabids; Rosales; Rosaceae; Rosoideae; Potentilleae; Potentilleae incertae sedis; *Potentilla*; *Potentilla anserina* (Linnaeus, 1753) (NCBI:txid57926).

## Background

**Figure 1.**
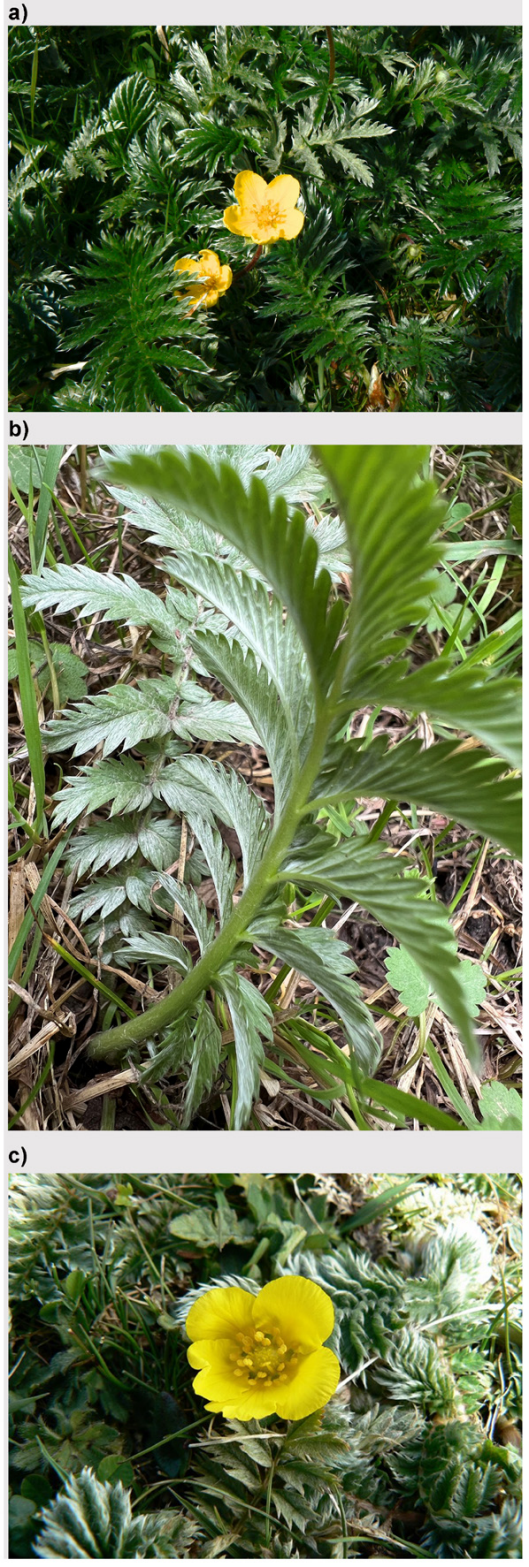
Example images of *Potentilla anserina* (not the sampled specimen) growing in Wytham Woods, Oxfordshire, showing ( **a**) the whole plant, ( **b**) a close-up view of the silvery leaves, and ( **c**) a close-up of the flower (photos A.R. Leitch).

*Potentilla anserina* L. (Rosaceae) ( Figure 1) is a wide-spread and common perennial across Britain and Ireland which can easily be spotted creeping along the ground in a diversity of habitats like meadows, grasslands, riversides, rough ground and roadsides. Its pinnate leaves are covered below in silver-grey hairs giving rise to its common name, silverweed.

Taxonomy of the genus *Potentilla* L. is contentious, and based on molecular phylogenetic studies using nuclear and plastid markers ( Eriksson *et al.*, 1998; Töpel *et al.*, 2011), it was shown that the genus in the traditional sense is polyphyletic, with diverse genera like *Alchemilla* L. and *Fragaria* L. embedded in it. *Potentilla* is sometimes treated in the broad sense to include these genera, but it is also often subdivided, with *Potentilla anserina* (and 70 related species) placed in the genus *Argentina* Hill [as *A. anserina* (L.) Rydb.]. Nevertheless, we here follow Stace (2010) to be consistent with the taxonomy used for all British and Irish vascular plants sampled by the Darwin Tree of Life. 

Globally, silverweed cinquefoil has an extensive native range from the Arctic regions of North America and Eurasia to the mountains of New Mexico and the Himalayas in Asia ( POWO, 2023; Rousi, 1965). It is tolerant of disturbance, pollution, inundation and drought. It is therefore not surprising that it is widespread and thriving across Britain and Ireland, with little change in its distribution over the last 60 years. It is also frequently naturalised outside its native range, including Argentina, Australia, Chile and New Zealand.

Many uses of *P. anserina* have been documented, giving rise to some of the local names associated with this species. For example, it has been called ‘traveller’s ease’, perhaps from reports that it was used by Roman soldiers as a botanical shoe insole to provide padding and absorb sweat on their long marches (possibly because the high starch content helps to absorb moisture). In addition, its highly starchy stolons often provided an important food source, especially during famine, giving rise to the name of ‘bread and butter’ in some parts of the UK. For thousands of years *P. anserina* has been used in traditional medicine to treat a wide diversity of symptoms – e.g. coughs, diarrhoea and viral infections ( Tomczyk & Latté, 2009) – with recent biochemical studies validating their use – e.g. the role of distinctive polysaccharides extracted from roots in relieving coughing ( Guo *et al.*, 2016) and the suppression of Hepatitis B growth by a specific triterpenoid saponin isolated from the rhizome ( Zhao *et al.*, 2008).

While tetraploid (2 *n* = 4 *x* = 28), pentaploid (2 *n* = 5 *x* = 34) and hexaploid (2 *n* = 6 *x* = 42) individuals of *P. anserina* have been reported, tetraploids are the most common across its native distribution ( Ockendon & Walters, 1970; Rice *et al.*, 2015), with a recent genomic analysis suggesting that the tetraploid is an ancient allopolyploid estimated to have formed *c*. 6.4 million years ago ( Gan *et al.*, 2021). While the ancestral genome donors are unclear, comparative sequence analysis suggests that one of the two parents may be *P. micrantha* Ramond ex DC. or a close relative ( Gan *et al.*, 2021). Fossil achenes are known from the Pleistocene and Pliocene of Europe ( Velichkevich & Zastawniak, 2003).

The in-depth genomic analyses of a tetraploid accession of *P. anserina*, made possible by the release of this high-quality chromosome level genome sequence, will enable further exploration of the evolution of this ancient polyploid genome. Analyses of the genomic networks underpinning the biochemical pathways, which have contributed to the rich diversity of metabolites, may also provide opportunities for bioprospecting to identify novel medicines.

## Genome sequence report

The genome was sequenced from a *Potentilla anserina* specimen collected from along the River Thames in Canbury Gardens, Kingston upon Thames (latitude 51.42, longitude –0.31). Using flow cytometry, the genome size (1C-value) was estimated to be 0.56 pg, equivalent to 550 Mb which is similar in size to a previous estimate by flow cytometry for an autotetraploid (2 *n* = 4 *x* = 48) cytotype ( Šmarda *et al.*, 2019) and gives an estimated haploid genome size comprising 7 chromosomes of 275 Mb. A total of 36-fold coverage in Pacific Biosciences single-molecule HiFi long reads and 61-fold coverage in 10X Genomics read clouds were generated. Primary assembly contigs were scaffolded with chromosome conformation Hi-C data. Manual assembly curation corrected eight missing joins or mis-joins and removed 12 haplotypic duplications, reducing the assembly length by 48.31% and the scaffold number by 48.43%, and increasing the scaffold N50 by 18.96%.

**Figure 2.**
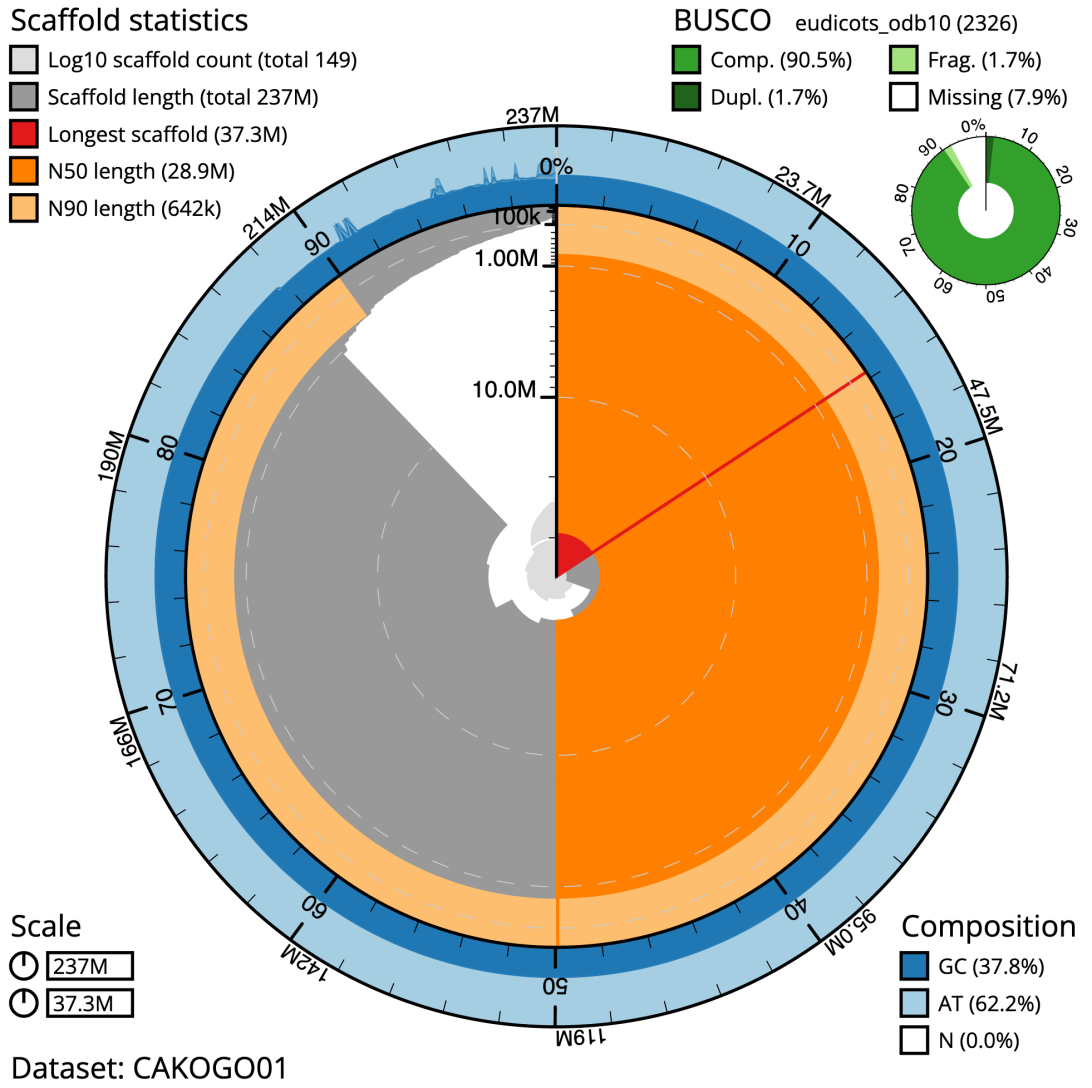
Genome assembly of *Potentilla anserina*, drPotAnse1.1: metrics. The BlobToolKit Snailplot shows N50 metrics and BUSCO gene completeness. The main plot is divided into 1,000 size-ordered bins around the circumference with each bin representing 0.1% of the 237,129,565 bp assembly. The distribution of scaffold lengths is shown in dark grey with the plot radius scaled to the longest scaffold present in the assembly (37,348,946 bp, shown in red). Orange and pale-orange arcs show the N50 and N90 scaffold lengths (28,859,254 and 641,888 bp), respectively. The pale grey spiral shows the cumulative scaffold count on a log scale with white scale lines showing successive orders of magnitude. The blue and pale-blue area around the outside of the plot shows the distribution of GC, AT and N percentages in the same bins as the inner plot. A summary of complete (Comp.), fragmented (Frag.), duplicated (Dupl.) and missing BUSCO genes in the eudicots_odb10 set is shown in the top right. An interactive version of this figure is available at https://blobtoolkit.genomehubs.org/view/drPotAnse1.1/dataset/CAKOGO01/snail.

The final haploid assembly has a total length of 237.1 Mb in 148 sequence scaffolds with a scaffold N50 of 28.9 Mb ( Table 1). Most (87.84%) of the assembly sequence was assigned to seven chromosomal-level scaffolds. Chromosome-scale scaffolds are named by synteny based on the genome assembly of *Fragaria nilgerrensis* Schltdl. ex J.Gay (eudicots) GCA_010134655.1 ( Figure 2– Figure 5; Table 2). The mitochondrial and plastid genomes were also assembled. The assembly has a BUSCO v5.3.2 ( Manni *et al.*, 2021) completeness of 90.5% (single 88.8%, duplicated 1.7%) using the eudicots_odb10 reference set ( *n* = 2,326). While not fully phased, the assembly deposited is of one haplotype. Contigs corresponding to the other similar three haplotype sets of the tetraploid have also been deposited.

**Table 1.** Genome data for *Potentilla anserina*, drPotAnse1.1.

Project accession data		
Assembly identifier	drPotAnse1.1.	
Species	*Potentilla anserina*	
Specimen	drPotAnse1	
NCBI taxonomy ID	57926	
BioProject	PRJEB46847	
BioSample ID	SAMEA7522052	
Isolate information	drPotAnse1: Leaf tissue	
Assembly metrics *		*Benchmark*
Consensus quality (QV)	55.3	*≥ 50*
*k*-mer completeness	99.99%	*≥ 95%*
BUSCO **	C:90.5%[S:88.8%,D:1.7%], F:1.7%,M:7.9%,n:2,326	*C ≥ 95%*
Percentage of assembly mapped to chromosomes	87.84%	*≥ 95%*
Sex chromosomes	N/A	*localised homologous* *pairs*
Organelles	Mitochondrial and plastid genomes assembled	*complete single alleles*
Raw data accessions		
PacificBiosciences SEQUEL II	ERR6808017	
10X Genomics Illumina	ERR6688615–ERR6688618	
Hi-C Illumina	ERR6688619	
PolyA RNA-Seq Illumina	ERR9435015	
Genome assembly		
Assembly accession	GCA_933775445.1	
*Accession of alternate haplotype*	GCA_933772775.1	
Span (Mb)	237.1	
Number of contigs	156	
Contig N50 length (Mb)	18.7	
Number of scaffolds	148	
Scaffold N50 length (Mb)	28.9	
Longest scaffold (Mb)	37.4	

* Assembly metric benchmarks are adapted from column VGP-2020 of “Table 1: Proposed standards and metrics for defining genome assembly quality” from Rhie *et al.* (2021). ** BUSCO scores based on the eudicots_odb10 BUSCO set using v5.3.2. C = complete [S = single copy, D = duplicated], F = fragmented, M = missing, n = number of orthologues in comparison. A full set of BUSCO scores is available at https://blobtoolkit.genomehubs.org/view/drPotAnse1.1/dataset/CAKOGO01/busco.

**Figure 3.**
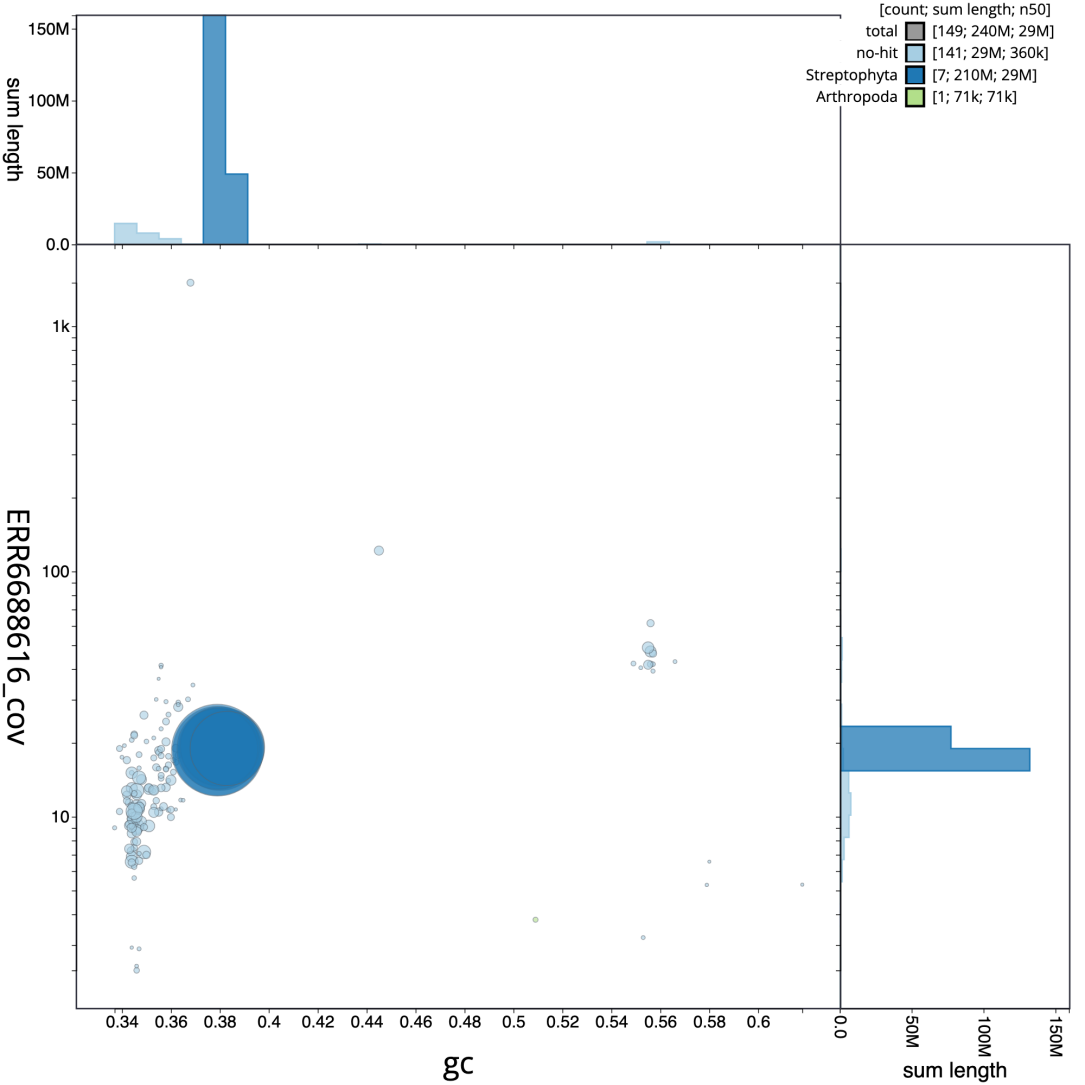
Genome assembly of *Potentilla anserina*, drPotAnse1.1: GC coverage. BlobToolKit GC-coverage plot. Scaffolds are coloured by phylum. Circles are sized in proportion to scaffold length. Histograms show the distribution of scaffold length sum along each axis. An interactive version of this figure is available at https://blobtoolkit.genomehubs.org/view/drPotAnse1.1/dataset/CAKOGO01/blob.

**Figure 4.**
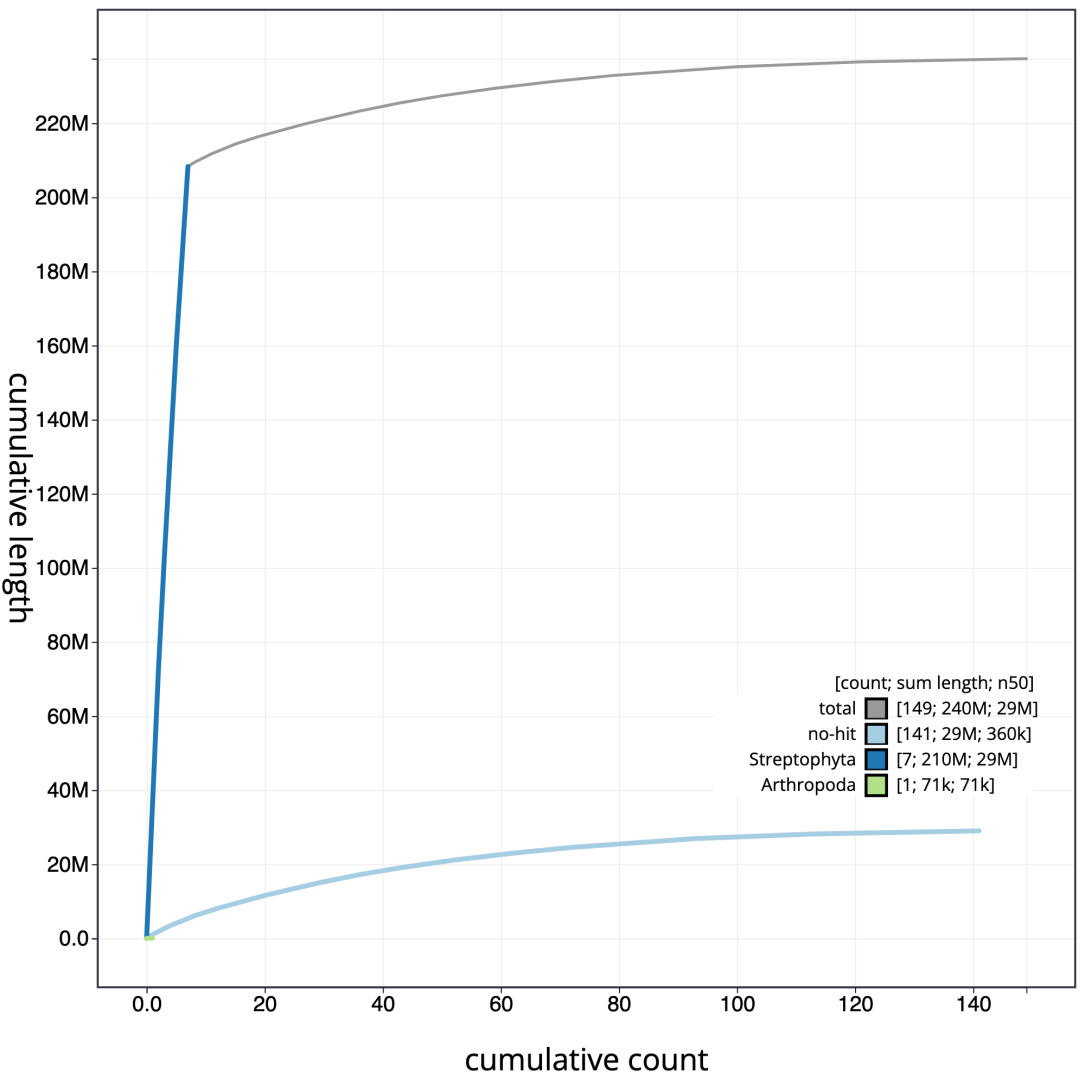
Genome assembly of *Potentilla anserina*, drPotAnse1.1: cumulative sequence. BlobToolKit cumulative sequence plot. The grey line shows cumulative length for all scaffolds. Coloured lines show cumulative lengths of scaffolds assigned to each phylum using the buscogenes taxrule. An interactive version of this figure is available at https://blobtoolkit.genomehubs.org/view/drPotAnse1.1/dataset/CAKOGO01/cumulative.

**Figure 5.**
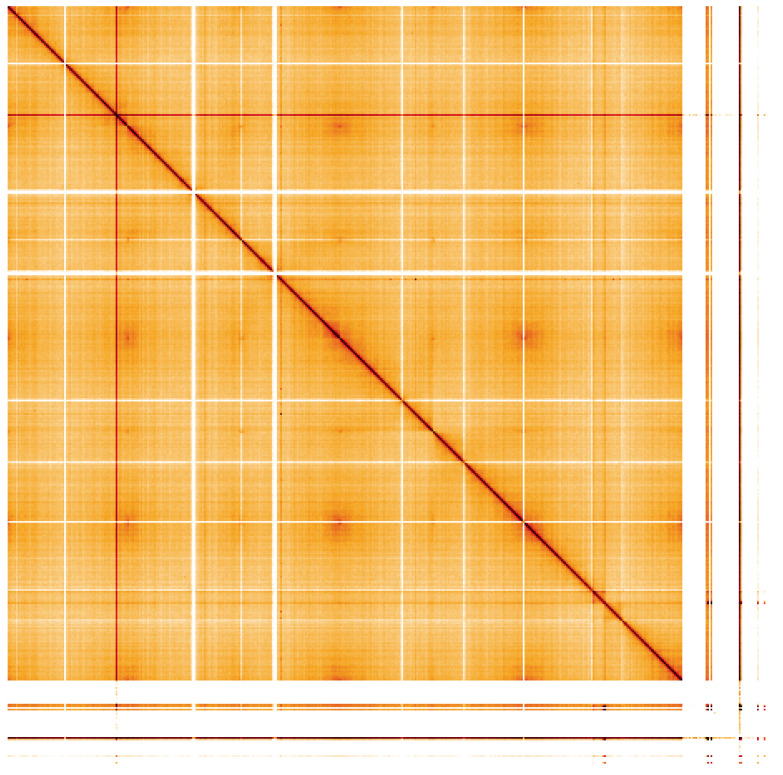
Genome assembly of *Potentilla anserina*, drPotAnse1.1: Hi-C contact map. Hi-C contact map of the drPotAnse1.1 assembly, visualised using HiGlass. An interactive version of this figure may be viewed at https://genome-note-higlass.tol.sanger.ac.uk/l/?d=XseUnFh8SLiNc1pwzZ5tXw.

**Table 2.** Chromosomal pseudomolecules in the genome assembly of *Potentilla anserina*, drPotAnse1.

INSDC accession	Chromosome	Size (Mb)	GC%
OW176981.1	1	24.72	38.3
OW176982.1	2	30.21	38
OW176983.1	3	35.14	37.9
OW176984.1	4	27.77	38
OW176985.1	5	28.86	37.9
OW176986.1	6	37.35	37.9
OW176987.1	7	24.26	38.3
OW176988.1	MT	0.29	44.5
OW176989.1	Pltd	0.16	36.8
-	-	28.66	36.3

## Methods

### Sample acquisition, genome size estimation and nucleic acid extraction

A *Potentilla anserina* specimen (drPotAnse1) was collected in Canbury Gardens, Kingston upon Thames, Surrey, UK (latitude 51.42, longitude –0.31) on 12 August 2020. The specimen was picked by hand by Maarten Christenhusz (Royal Botanic Gardens, Kew) collection number 9040, and identified by the same person. The specimen was preserved at –80°C.

The genome size was estimated by flow cytometry using the fluorochrome propidium iodide and following the ‘one-step’ method outlined in ( Pellicer *et al.*, 2021). Specifically for this species, the General Purpose Buffer (GPB) supplemented with 3% PVP and 0.08% (v/v) beta-mercaptoethanol was used for isolation of nuclei ( Loureiro *et al.*, 2007), and the internal calibration standard was *Petroselinum crispum* ‘Champion Moss Curled’ with an assumed 1C-value of 2.20 Gb ( Obermayer *et al.*, 2002).

DNA was extracted at the Tree of Life laboratory, Wellcome Sanger Institute (WSI). The drPotAnse1 sample was weighed and dissected on dry ice with tissue set aside for Hi-C sequencing. Leaf tissue was cryogenically disrupted to a fine powder using a Covaris cryoPREP Automated Dry Pulveriser, receiving multiple impacts. High molecular weight (HMW) DNA was extracted using the Qiagen Plant MagAttract HMW DNA extraction kit. Low molecular weight DNA was removed from a 20 ng aliquot of extracted DNA using the 0.8X AMpure XP purification kit prior to 10X Chromium sequencing; a minimum of 50 ng DNA was submitted for 10X sequencing. HMW DNA was sheared into an average fragment size of 12–20 kb in a Megaruptor 3 system with speed setting 30. Sheared DNA was purified by solid-phase reversible immobilisation using AMPure PB beads with a 1.8X ratio of beads to sample to remove the shorter fragments and concentrate the DNA sample. The concentration of the sheared and purified DNA was assessed using a Nanodrop spectrophotometer and Qubit Fluorometer and Qubit dsDNA High Sensitivity Assay kit. Fragment size distribution was evaluated by running the sample on the FemtoPulse system.

RNA was extracted from the leaf tissue of drPotAnse1 in the Tree of Life Laboratory at the WSI using TRIzol, according to the manufacturer’s instructions. RNA was then eluted in 50 μl RNAse-free water and its concentration assessed using a Nanodrop spectrophotometer and Qubit Fluorometer using the Qubit RNA Broad-Range (BR) Assay kit. Analysis of the integrity of the RNA was done using Agilent RNA 6000 Pico Kit and Eukaryotic Total RNA assay.

### Sequencing

Pacific Biosciences HiFi circular consensus and 10X Genomics read cloud DNA sequencing libraries were constructed according to the manufacturers’ instructions. Poly(A) RNA-Seq libraries were constructed using the NEB Ultra II RNA Library Prep kit. DNA and RNA sequencing was performed by the Scientific Operations core at the WSI on Pacific Biosciences SEQUEL II (HiFi), Illumina HiSeq 4000 (RNA-Seq) and Illumina NovaSeq 6000 (10X) instruments. Hi-C data were also generated from tissue of drPotAnse1 using the Arima v2 kit and sequenced on the Illumina NovaSeq 6000 instrument.

### Genome assembly, curation and evaluation

Assembly was carried out with Hifiasm ( Cheng *et al.*, 2021) and haplotypic duplication was identified and removed with purge_dups ( Guan *et al.*, 2020). One round of polishing was performed by aligning 10X Genomics read data to the assembly with Long Ranger ALIGN, calling variants with FreeBayes ( Garrison & Marth, 2012). The assembly was then scaffolded with Hi-C data ( Rao *et al.*, 2014) using SALSA2 ( Ghurye *et al.*, 2019). The assembly was checked for contamination as described previously ( Howe *et al.*, 2021). Manual curation was performed using HiGlass ( Kerpedjiev *et al.*, 2018) and Pretext ( Harry, 2022). The mitochondrial and chloroplast genomes were assembled using MBG ( Rautiainen & Marschall, 2021) from PacBio HiFi reads mapping to related genomes. A representative circular sequence was selected for each from the graphs based on read coverage.

A Hi-C map for the final assembly was produced using bwa-mem2 ( Vasimuddin *et al.*, 2019) in the Cooler file format ( Abdennur & Mirny, 2020). To assess the assembly metrics, the *k*-mer completeness and QV consensus quality values were calculated in Merqury ( Rhie *et al.*, 2020). This work was done using Nextflow ( Di Tommaso *et al.*, 2017) DSL2 pipelines “sanger-tol/readmapping” ( Surana *et al.*, 2023a) and “sanger-tol/genomenote” ( Surana *et al.*, 2023b). The genome was analysed within the BlobToolKit environment ( Challis *et al.*, 2020) and BUSCO scores ( Manni *et al.*, 2021; Simão *et al.*, 2015) were calculated.

Table 3 contains a list of relevant software tool versions and sources.

**Table 3.** Software tools: versions and sources.

Software tool	Version	Source
BlobToolKit	3.4.0	https://github.com/blobtoolkit/blobtoolkit
BUSCO	5.3.2	https://gitlab.com/ezlab/busco
FreeBayes	1.3.1-17-gaa2ace8	https://github.com/freebayes/freebayes
Hifiasm	0.15.3	https://github.com/chhylp123/hifiasm
HiGlass	1.11.6	https://github.com/higlass/higlass
Long Ranger ALIGN	2.2.2	https://support.10xgenomics.com/genome-exome/software/pipelines/ latest/advanced/other-pipelines
Merqury	MerquryFK	https://github.com/thegenemyers/MERQURY.FK
MitoHiFi	2	https://github.com/marcelauliano/MitoHiFi
PretextView	0.2	https://github.com/wtsi-hpag/PretextView
purge_dups	1.2.3	https://github.com/dfguan/purge_dups
SALSA	2.2	https://github.com/salsa-rs/salsa
sanger-tol/genomenote	v1.0	https://github.com/sanger-tol/genomenote
sanger-tol/readmapping	1.1.0	https://github.com/sanger-tol/readmapping/tree/1.1.0

### Wellcome Sanger Institute – Legal and Governance

The materials that have contributed to this genome note have been supplied by a Darwin Tree of Life Partner. The submission of materials by a Darwin Tree of Life Partner is subject to the **‘Darwin Tree of Life Project Sampling Code of Practice’**, which can be found in full on the Darwin Tree of Life website here. By agreeing with and signing up to the Sampling Code of Practice, the Darwin Tree of Life Partner agrees they will meet the legal and ethical requirements and standards set out within this document in respect of all samples acquired for, and supplied to, the Darwin Tree of Life Project.

Further, the Wellcome Sanger Institute employs a process whereby due diligence is carried out proportionate to the nature of the materials themselves, and the circumstances under which they have been/are to be collected and provided for use. The purpose of this is to address and mitigate any potential legal and/or ethical implications of receipt and use of the materials as part of the research project, and to ensure that in doing so we align with best practice wherever possible. The overarching areas of consideration are:

•   Ethical review of provenance and sourcing of the material

•   Legality of collection, transfer and use (national and international) 

Each transfer of samples is further undertaken according to a Research Collaboration Agreement or Material Transfer Agreement entered into by the Darwin Tree of Life Partner, Genome Research Limited (operating as the Wellcome Sanger Institute), and in some circumstances other Darwin Tree of Life collaborators.

## Data Availability

European Nucleotide Archive: *Potentilla anserina*. Accession number PRJEB46847; https://identifiers.org/ena.embl/PRJEB46847. ( Wellcome Sanger Institute, 2022) The genome sequence is released openly for reuse. The *Potentilla anserina* genome sequencing initiative is part of the Darwin Tree of Life (DToL) project. All raw sequence data and the assembly have been deposited in INSDC databases. The genome will be annotated using available RNA-Seq data and presented through the Ensembl pipeline at the European Bioinformatics Institute. Raw data and assembly accession identifiers are reported in Table 1.

## Author information

Members of the Royal Botanic Gardens Kew Genome Acquisition Lab are listed here: https://doi.org/10.5281/zenodo.4786680.

Members of the Plant Genome Sizing collective are listed here: https://doi.org/10.5281/zenodo.7994306.

Members of the Darwin Tree of Life Barcoding collective are listed here: https://doi.org/10.5281/zenodo.4893703.

Members of the Wellcome Sanger Institute Tree of Life programme are listed here: https://doi.org/10.5281/zenodo.4783585.

Members of Wellcome Sanger Institute Scientific Operations: DNA Pipelines collective are listed here: https://doi.org/10.5281/zenodo.4790455.

Members of the Tree of Life Core Informatics collective are listed here: https://doi.org/10.5281/zenodo.5013541.
