# Supplementary material for: The genome sequence of the silverweed cinquefoil, Potentilla anserina L., 1753
Source: Wellcome Open Res. 2023 Oct 13;8:464. [Version 1] doi: 10.12688/wellcomeopenres.19908.1 (PMC11162522; doi:10.12688/wellcomeopenres.19908.1)
Supplement: Peer review history

# Reviewer report: Reviewer response for version 1

**DOI:** 10.21956/wellcomeopenres.22044.r83464

**Published:** 2024-06-08

**Authors:** Diego F. Morales-Briones

**Affiliations:** 1 Ludwig Maximilian University, Munich, Germany

This Data Note provides the genome (nuclear, mitochondrial, and chloroplast) assembly of Potentilla anserina. The nuclear haploid genome comprised 237 megabases, mostly assigned to seven chromosomes.

 Overall, the manuscript is well-written and provides all the necessary information. Still, a brief comparison to a previous genome sequence report (Gan et al., 2021) of the same species can be included. Providing assembly metrics for the Gan et al. (2021) in Table 1 should be sufficient.

Are sufficient details of methods and materials provided to allow replication by others?

Yes

Is the rationale for creating the dataset(s) clearly described?

Yes

Are the datasets clearly presented in a useable and accessible format?

Yes

Are the protocols appropriate and is the work technically sound?

Yes

Reviewer Expertise:

Phylogenomics and Plant Systematics

I confirm that I have read this submission and believe that I have an appropriate level of expertise to confirm that it is of an acceptable scientific standard.

# Reviewer report: Reviewer response for version 1

**DOI:** 10.21956/wellcomeopenres.22044.r83457

**Published:** 2024-05-30

**Authors:** Xin Qiao

**Affiliations:** 1 Nanjing Agricultural University, Nanjing, China

This article reports a genome assembly of *Potentilla anserina*. About 88% of the assembly sequence was assigned to seven chromosomes.  This genome provides a new addition for comparative genomic analyses of Rosaceae species and polyploidy evolution. I am happy to see the publication of this article.

 In a previous study, *Potentilla anserina *was inferred to be a allotetraploid and two sub genomes A and B were assembled (Gan et al., 2021). However, in this study, only one haploid was assembled. Please clarify the difference in genome assembly between this study and Gan's report.

Are sufficient details of methods and materials provided to allow replication by others?

Yes

Is the rationale for creating the dataset(s) clearly described?

Yes

Are the datasets clearly presented in a useable and accessible format?

Yes

Are the protocols appropriate and is the work technically sound?

Yes

Reviewer Expertise:

comparative genomics

I confirm that I have read this submission and believe that I have an appropriate level of expertise to confirm that it is of an acceptable scientific standard.
